# TRPV1 activity and substance P release are required for corneal cold nociception

**DOI:** 10.1038/s41467-019-13536-0

**Published:** 2019-12-12

**Authors:** Fengxian Li, Weishan Yang, Haowu Jiang, Changxiong Guo, Andrew J. W. Huang, Hongzhen Hu, Qin Liu

**Affiliations:** 1 Department of Anesthesiology, Center for the Study of Itch and Sensory Disorders, Washington University Pain Center, Washington University School of Medicine, St. Louis, MO USA; 2 Department of Anesthesiology, Zhujiang Hospital of Southern Medical University, Guangdong, China; 3 Department of Ophthalmology & Visual Sciences, Washington University School of Medicine, St. Louis, MO USA

**Keywords:** Sensory processing, Corneal diseases

## Abstract

As a protective mechanism, the cornea is sensitive to noxious stimuli. Here, we show that in mice, a high proportion of corneal TRPM8^+^ cold-sensing fibers express the heat-sensitive TRPV1 channel. Despite its insensitivity to cold, TRPV1 enhances membrane potential changes and electrical firing of TRPM8^+^ neurons in response to cold stimulation. This elevated neuronal excitability leads to augmented ocular cold nociception in mice. In a model of dry eye disease, the expression of TRPV1 in TRPM8^+^ cold-sensing fibers is increased, and results in severe cold allodynia. Overexpression of TRPV1 in TRPM8^+^ sensory neurons leads to cold allodynia in both corneal and non-corneal tissues without affecting their thermal sensitivity. TRPV1-dependent neuronal sensitization facilitates the release of the neuropeptide substance P from TRPM8^+^ cold-sensing neurons to signal nociception in response to cold. Our study identifies a mechanism underlying corneal cold nociception and suggests a potential target for the treatment of ocular pain.

## Abstract (web summary)

The eye shows protective responses to noxious stimuli including cold. Here, the authors show that TRPV1, found co-expressed on TRPM8 + fibres in the cornea, is necessary for cold nociception in the eye.

## Introduction

Cold allodynia (pain elicited by a normally innocuous cold stimulus) is a common symptom of fibromyalgia, peripheral neuropathy, tissue injury/inflammation, and sensitive teeth. Although most tissues lack cold hypersensitivity under normal conditions, human psychophysical studies revealed that: in the cornea, stimulation with low temperatures was perceived as a cooling sensation with an irritative component^1^. The cornea is one of the most unique tissues in the human body. It is richly supplied by primary sensory fibers from the ophthalmic division (V1) of the trigeminal ganglia (TG). Pleasant cooling that is innocuous to other parts of human body can induce significant irritation in the cornea^1^, which resembles pathological cold allodynia. However, it is unclear whether corneal cold nociception shares a common mechanism with pathological cold allodynia.

Studies have revealed that Transient Receptor Potential Channel M8 (TRPM8) functions as the cold receptor in the cornea and other tissues^2^. TRPM8 is widely expressed in corneal afferent fibers^3,4^, and regulates basal tearing by sensing temperature drops induced by tear evaporation^2^. However, it remains contentious whether TRPM8-expressing sensory fibers mediate cold nociception in the eye^2,5^, even though TRPM8 does mediate cold allodynia in the skin^6,7^.

Cold nociception and allodynia are frequently characterized by an irritative, burning sensation in the cornea or under pathologic conditions in humans^1^. The burning sensation is usually mediated by heat-sensitive channels, such as TRPV1. TRPV1 is a cationic channel sensitive to capsaicin, protons and noxious heat (but not cold), and mediates burning pain evoked by each of these stimuli^8,9^. Its co-expression with TRPM8 is minimal in the skin sensory fibers^10–12^. However, 65% of cold endings respond to capsaicin in the mouse cornea^2^, suggesting a high overlap between TRPM8 and TRPV1 in the corneal sensory fibers. In this study, we confirmed that a substantial proportion of corneal TRPM8^+^ fibers express TRPV1. Strikingly, this co-expression of TRPV1 with TRPM8 is both necessary and sufficient for cold nociception and allodynia in the cornea, but does not alter the corneal thermal sensitivity. As TRPV1 has been shown to display tonic activity at body temperature^13,14^, the upregulation of TRPV1 enhances excitability of TRPM8^+^ sensory neurons and promotes the neuronal release of the neuropeptide substance P to signal cold nociception. This study provides a molecular mechanism underlying the cold nociception of the cornea and pathological cold allodynia, and sheds light on the molecules, cells and circuits for cold percepts.

## Results

### TRPM8 mediates ocular cold nociception

**Fig. 1:**
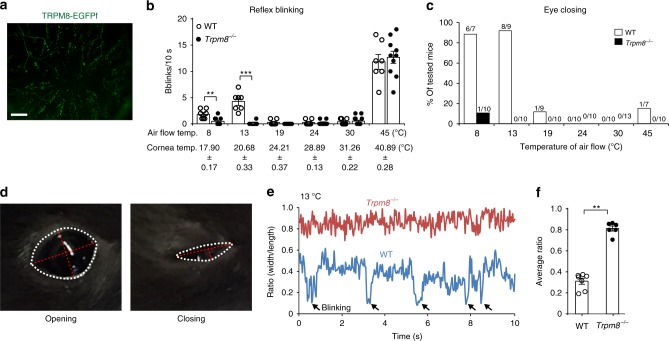
TRPM8 mediates cold-induced ocular nociception. **a** Representative image showing that TRPM8-expressing sensory fibers (green) densely innervate the cornea as revealed by TRPM8-EGFPf in the whole-mount cornea from *Trpm8*^*EGFPf/+*^ mice. **b**, **c** The reflex blinking and eye closing responses to air flow (0.5 L/min) at different temperature in WT (*n* = 7–10) and *Trpm8*^*−/−*^ (*n* = 10–13) mice. **d** Representative video frames showing the opening and closing of the mouse eye (outlined by white dash lines). Red dash lines indicate the width and length of the palpebral fissure. **e** The representative width/length ratio changes of WT and *Trpm8*^*−/*−^ mice in response to cold air (13 °C). Black arrows indicate reflex blinking responses. **f** The average width/length ratio of WT mice (*n* = 7) is significantly lower than that of *Trpm8*^*−/−*^ mice (*n* = 6) in response to cold air (13 °C). Data are expressed as mean ± s.e.m. Statistical analysis by two tailed Student’s *t*-test. **P < 0.01; ***P < 0.001. All images shown are representative of three independent experiments using tissues from at least three different mice. Scale bar: 100 µm. Source data are provided as a Source Data file.

Utilizing *Trpm8*^*EGFPf/+*^ mice^10^, we found that TRPM8^+^ sensory fibers branch extensively and terminate in the superficial layers of the corneal epithelium (Fig. 1a). To determine the role of TRPM8 in cold nociception of the cornea, we tested the response of TRPM8-deficient mice to cold by exposing their eyes to gentle air flow at different temperatures. The gentle air flow (0.5 L/min) alone does not produce noxious mechanical stimulus to the cornea, as evidenced by a lack of behavioral responses to air flow at 24 °C (room temperature) and 30 °C in mice (Fig. 1b, c). However, the air flow at low and high temperatures effectively changes the temperature of the corneal surface (Fig. 1b; Supplementary Fig. 1) and elicits reflex blinking and even eye closing in mice (Fig. 1b, c). These ocular responses were also observed after ocular challenge with a pain-inducing compound capsaicin (Supplementary Fig. 2), suggesting that reflex blinking and eye closing are indicative behavior of ocular nociception. TRPM8-deficiency abolished both reflex blinking and eye closing responses to cold but not to heat (Fig. 1b, c), indicating that TRPM8 mediates ocular cold nociception.

In addition to TRPM8 channel, TRPA1 has been shown to act as a cold sensor^15^. To determine the function of TRPA1 in ocular cold nociception, we tested the ocular responses of *Trpa1*^*−/−*^ mice to cold. No significant difference was found in cold-induced reflex blinking or eye closing responses between *Trpa1*^*−/−*^ and wild type (WT) mice (Supplementary Fig. 3), arguing against the involvement of TRPA1 in corneal cold nociception.

Both reflex blinking and eye closing reduce the corneal exposure to noxious stimuli. By monitoring changes in the width/length ratio of the palpebral fissure of the eye (Fig. 1d)^16^, we found no difference between WT and *Trpm8*^*−/−*^ mice in their basal ratio at room temperature (Supplementary Fig. 4a). However, the average ratio of *Trpm8*^*−/−*^ mice in response to cold is significantly higher than that of WT mice and is indistinguishable from their basal ratio (Fig. 1e, f; Supplementary Fig. 4), indicating that TRPM8-deficiency impairs cold nociception.

In addition to cold, cryosim-3 (1-diisopropylphosphorylnonane)^17^ was utilized to activate TRPM8. As a TRPM8 agonist, cryosim-3 is more potent and specific for TRPM8 than menthol (a classic TRPM8 agonist)^17^. Our previous study has shown that application of cryosim-3 to the eyelid skin induces tearing and alleviates dry eye symptoms in dry eye patients^17^. Although cryosim-3 did not evoke nociception when applied to the eyelid skin^17^, it induced reflex blinking and eye closing in a dose-dependent manner when applied onto the cornea (Supplementary Fig. 5), suggesting that activation of TRPM8 in the cornea generates ocular nociception. Notably, eye closing was evoked only by high doses of cryosim-3 (Supplementary Fig. 5b), indicating that eye closing is a nocifensive behavioral response to more intense chemical stimuli. Correlating well with this, eye closing was evoked by more intense cold stimuli (8 and 13 °C), but not by moderate cold (19 °C, Fig. 1c).

### A high proportion of corneal TRPM8^+^ fibers express TRPV1

**Fig. 2:**
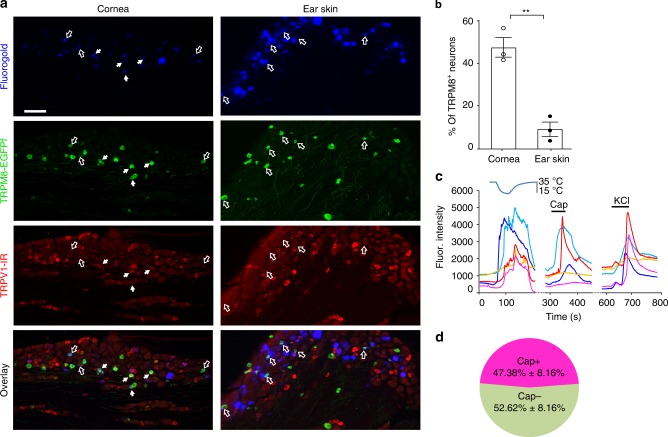
TRPV1 is co-expressed with TRPM8 in corneal sensory neurons. **a** Representative images showing a higher overlap between TRPM8-EGFPf and TRPV1-immunoreactivity (IR) in cornea-projecting neurons (retrogradely labeled by fluorogold from the cornea) than in skin-projecting neurons (retrogradely labeled by fluorogold from the ear skin). White arrows indicate fluorogold^+^/TRPM8^+^/TRPV1^+^ neurons, whereas hollow arrows indicate fluorogold^+^/TRPM8^+^/TRPV1^−^ neurons. **b** Quantification of the co-expression of TRPV1 with TRPM8 in sensory neurons innervating the cornea and ear skin, respectively (*n* = 3 mice/group). **c** Representative neuronal calcium responses to cold, capsaicin (10 µM), and KCl (100 mM) within the cornea-projection area in the ophthalmic division of the trigeminal ganglion explants from *Pirt*^*GCaMP3/+*^ mice in which a calcium indicator GCaMP3 was expressed in primary sensory neurons. **d** Quantification of capsaicin responses in 56 cold-sensitive neurons located within the cornea-projection area of four trigeminal ganglia from three *Pirt*^*GCaMP3/+*^ mice. Cap: capsaicin. Data are expressed as mean ± s.e.m. Statistical analysis by two tailed Student’s *t*-test. **P < 0.01. All images shown are representative of three independent experiments using tissues from at least three different mice. Scale bars in **a** 100 μm. Source data are provided as a Source Data file.

The indispensable role of TRPM8 in ocular cold nociception raises the question of why activation of TRPM8 by innocuous cold induces irritation/discomfort in the cornea, rather than a pleasant cooling sensation as in the skin or other parts of the body. Because ocular cold nociception is characterized by an irritative, burning component^1^, we hypothesize that the burning component of cold nociception is mediated by heat-sensitive channels such as TRPV1. To test this, we examined whether TRPV1 is co-expressed with TRPM8 in sensory neurons innervating the cornea. Retrogradely labeled corneal neurons were all located in a restricted area of the ophthalmic (V1) division of TG (Fig. 2a). Interestingly, ~47% of labeled TRPM8^+^ neurons displayed immunoreactivity for TRPV1 (Fig. 2a, b). By contrast, minimal co-expression of TRPM8 and TRPV1 was noted in sensory neurons that innervate the ear skin (Fig. 2a, b). This uniquely high overlap between TRPM8 and TRPV1 in cornea-projecting sensory neurons was further confirmed by calcium imaging. Utilizing *Pirt*^*GCaMP3/+*^ mice in which a calcium indicator GCaMP3 was expressed in primary sensory neurons^18^, we conducted ex vivo whole-ganglion calcium imaging. Consistent with the immunostaining results, we found that approximately half of cold-sensing neurons responded to TRPV1 agonist capsaicin in the cornea-projecting area within the ophthalmic division of the TG explants (Fig. 2c, d).

### TRPV1 enhances the responsiveness of TRPM8^+^ neurons to cold

**Fig. 3:**
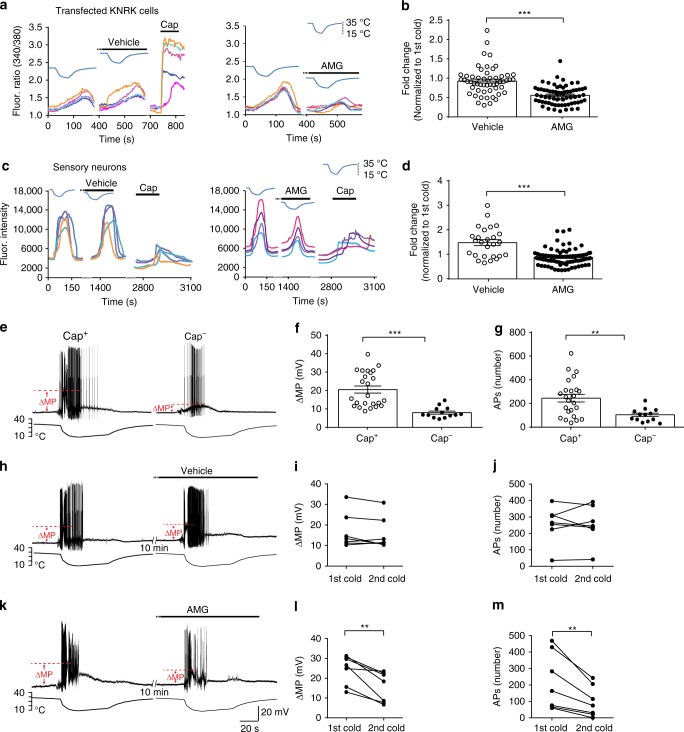
TRPV1 enhances the responsiveness of TRPM8-expressing cells to cold. **a**, **b** TRPV1 antagonist AMG9810 (AMG, 300 nM, 3 min pre-treatment before cold stimulation) attenuated calcium responses to cold in transfected KNRK cells that co-express TRPM8 and TRPV1. As a control, the vehicle treatment did not change their response to cold or capsaicin (Cap, 1 µM). Cold stimulation was generated by bath temperature drop from 32 °C to 15 °C. Each dot in **b** represents a KNRK cell that co-expresses TRPM8 and TRPV1. **c** Representative calcium transients of sensory neurons to cold stimuli after 10 min pre-treatment of vehicle (0.0006% DMSO in calcium imaging buffer) or AMG9810 (300 nM). Imaged neurons were located within the cornea-projection area in the ophthalmic division of the whole trigeminal ganglion explants (*n* = 7) from *Pirt*^*GCaMP3/+*^ transgenic mice, in which a calcium indicator GCaMP3 was expressed in primary sensory neurons. The expression of TRPV1 in tested neurons was determined by their responses to capsaicin (10 µM). **d** Quantification of calcium responses of trigeminal neurons to cold. Each dot represents one cold-sensing neuron. **e**–**g** Cold treatments elicited greater membrane potential changes (ΔMP) and more action potentials (APs) in a subset of TRPM8^EGFPf/+^ sensory neurons that are sensitive to capsaicin (1 µM, identified by calcium imaging) than those insensitive to capsaicin. **h**–**j** Repeated cold treatments did not desensitize TRPM8^EGFPf/+^; TRPV1^+^ sensory neurons in the vehicle control group, as shown by stable membrane potential changes and neuronal firing. **k**–**m** Application of TRPV1 antagonist AMG9810 (300 nM, 10 min pre-treatment before cold stimulation) effectively suppressed depolarization and neuronal firing in capsaicin-sensitive TRPM8^EGFPf/+^ neurons upon subsequent cold treatment. Data are expressed as mean ± s.e.m. Statistical analysis by two tailed Student’s *t*-test. **P < 0.01; ***P < 0.001. Source data are provided as a Source Data file.

The finding of TRPV1 expression in a large proportion of corneal TRPM8^+^ neurons prompted us to examine whether TRPV1 regulates the response of TRPM8^+^ cells to cold. TRPM8 and TRPV1 were co-expressed in heterologous KNRK cells. Pharmacological blockade of TRPV1 using AMG9810 (a potent and selective TRPV1 antagonist^19,20^) significantly decreased the calcium responses of the transfected cells to cold (Fig. 3a, b; Supplementary Fig. 6), suggesting that the expression of TRPV1 enhances the responsiveness of TRPM8^+^ cells to cold. We further examined the modulatory role of TRPV1 in TG sensory neurons. AMG9810 effectively reduced the amplitude of calcium responses to cold in ~48% of cold-sensing neurons that project to the cornea (Fig. 3c, d), correlating well with the proportion of TRPV1-expression or capsaicin sensitivity in TRPM8^+^ neurons that innervate the cornea.

We next examined whether the expression of TRPV1 enhances membrane depolarization and promotes action potential firing. In *Trpm8*^*EGFPf/+*^ mice, all capsaicin-sensitive TRPM8^+^ TG neurons (identified by calcium imaging) displayed greater membrane depolarization and fired more action potentials upon cold treatments than those insensitive to capsaicin (Fig. 3e–g), suggesting that the expression of TRPV1 promotes neuronal excitability to cold. Indeed, AMG9810 treatment significantly reduced the membrane depolarization and hence action potential firing in capsaicin-sensitive TRPM8^+^ neurons (Fig. 3k–m). As a control, vehicle treatment did not alter the membrane depolarization or action potential firing (Fig. 3h–j). These studies support an indispensable role of TRPV1 in potentiating the response of TRPM8^+^ neurons to cold.

### TRPV1 is required for TRPM8-mediated cornea cold nociception

**Fig. 4:**
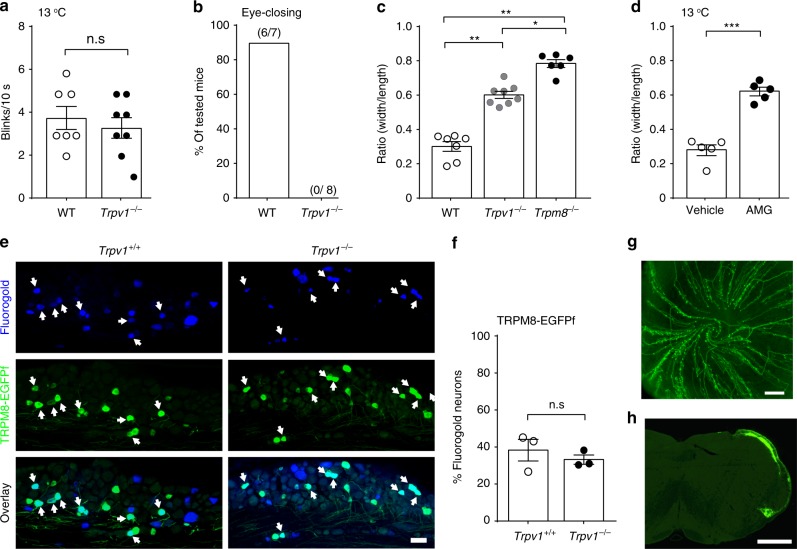
TRPV1 is required for ocular cold nociception. **a**, **b** Although the reflex blinking response to cold was not altered in *Trpv1*^*−/*−^ mice (*n* = 8), their eye closing responses were eliminated, compared with WT controls (*n* = 7). **c** The ocular width/length ratio of *Trpv1*^*−/−*^ mice (*n* = 8) in response to cold (13 °C) was significantly greater than that of WT mice (*n* = 7), but lower than that of *Trpm8*^−*/−*^ mice (*n* = 6). **d** The ocular width/length ratio in response to cold (13 °C) was increased in WT mice pre-treated with TRPV1 antagonist AMG9810 (0.2 nmol in 2 μL, 10 min before cold challenge; *n* = 5) compared with controls pre-treated with the vehicle (0.1% Tween-80 in saline; *n* = 5). **e**, **f** Representative images and group analysis show that TRPV1-deficiency did not impair the survival of corneal TRPM8-EGFPf neurons (retrogradely labeled by fluorogold from the cornea) in *Trpv1*^*−/−*^; *Trpm8*^*EGFPf/+*^ mice (*n* = 3), compared with control *Trpv1*^*+/+*^; *Trpm8*^*EGFPf/+*^ mice (*n* = 3). **g** Representative image showing that TRPM8-EGFPf sensory fibers (green) densely innervate the whole-mount cornea from *Trpv1*^*-/-*^; *Trpm8*^*EGFPf/+*^ mice. **h** Representative image showing the central projection of TRPM8-EGFPf fibers within the cornea-projection region in the spinal trigeminal nucleus of *Trpv1*^*−/−*^; *Trpm8*^*EGFPf/+*^ mice. Data are expressed as mean ± s.e.m. Statistical analysis by one-way ANOVA and two tailed Student’s *t*-test. n.s. not significant; *P < 0.05; **P < 0.01; ***P < 0.001. All images shown are representative of three independent experiments using tissues from at least three different mice. Scale bars in **e**, **g**, **h** 50, 100, and 250 μm, respectively. Source data are provided as a Source Data file.

The discovery that TRPV1 promotes excitability of TRPM8^+^ neurons raises the question of whether TRPV1 facilitates cold nociception of the cornea. Although TRPV1 is not activated by cold^21^, we found that *Trpv1*^*−/−*^ mice completely lacked the eye closing response to cold. In contrast, their reflex blinking response was not significantly reduced (Fig. 4a-c), suggesting that TRPV1 is required to produce intense nocifensive behavioral response. Correlating well with this result, pharmacological blockade of TRPV1 in the cornea using AMG9810 abolished the eye closing response to cold (13 °C) and increased the width/length ratio to the extent similar to that of TRPV1-deficient mice (Fig. 4d). These studies demonstrate that TRPV1 facilitates TRPM8-mediated cold nociception in the cornea. Of note, the nociceptive deficit of *Trpv1*^*−/−*^ mice to cold is not due to a poor survival of corneal TRPM8^+^ sensory neurons (Fig. 4e, f) or their defective projections to the cornea or brainstem (Fig. 4g, h).

### TRPV1 is required for dry eye-induced cold allodynia

**Fig. 5:**
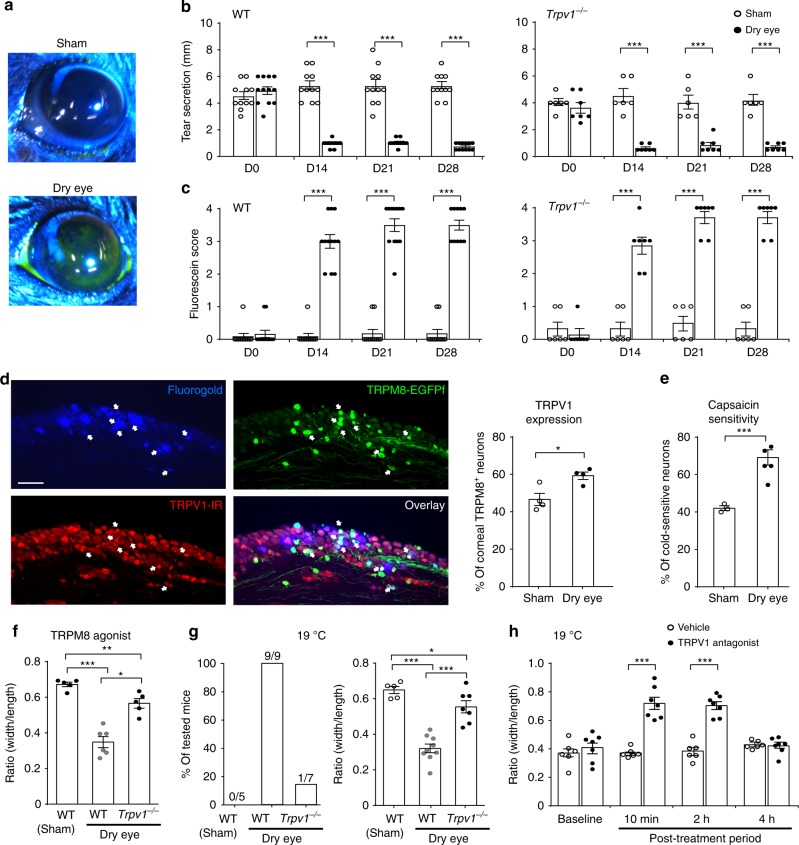
TRPV1 is required for dry eye-induced cold allodynia. **a** Representative images showing dry eye-associated corneal abrasion as revealed under a cobalt blue light after fluorescein staining. **b**, **c** *Trpv1*^*−/−*^ mice (*n* = 7) display normal basal tear secretion (D0) and similar extents of reduced tear secretion and intensified corneal epithelial erosions as control WT mice (*n* = 12) after surgical removal of exorbital lacrimal glands. Sham-operated WT (*n* = 11) or *Trpv1*^−*/*−^ mice (*n* = 6) do not show reduced tear secretion or corneal abrasion. **d** Representative images and group analysis show that the expression of TRPV1 was significantly increased among corneal TRPM8-EGFPf neurons (retrogradely labeled by fluorogold) in dry eye *Trpm8*^*EGFPf/+*^ mice (*n* = 4), compared with sham-operated mice (*n* = 4). White arrows indicate fluorogold^+^/TRPM8^+^/TRPV1^+^ neurons. **e** The proportion of cold-sensitive neurons that display capsaicin sensitivity was significantly greater in the dry eye group (*n* = 5), compared with the sham group (*n* = 3). **f** TRPM8-agonist cryosim-3 (0.025 nmol in 1 μL) elicited ocular nociception in dry eye WT mice (*n* = 6), but not in sham-operated WT mice (*n* = 5). *Trpv1*^*−/−*^ mice (*n* = 5) displayed significantly attenuated ocular nociception induced by cryosim-3 under dry eye conditions. **g** Innocuous cold (19 °C) elicited ocular nociception-associated eye closing in dry eye WT mice (*n* = 9), but not in sham-operated WT mice (*n* = 5). The eye closing response was significantly reduced in dry eye *Trpv1*^*−/−*^ mice (n = 7). **h** The effects of the TRPV1 antagonist AMG9810 (0.2 nmol in 2 μL) on ocular cold allodynia of dry eye WT mice (*n* = 7) at different post-treatment time points, compared with vehicle-treated control mice (*n* = 6). All the experiments in **d**–**h** were done four weeks after dry eye surgery. Data are expressed as mean ± s.e.m. Statistical analysis by one-way ANOVA and two tailed Student’s *t*-test. *P < 0.05; **P < 0.01; ***P < 0.001. All images shown are representative of three independent experiments using tissues from at least three different mice. Scale bar: 100 μm. Source data are provided as a Source Data file.

Under dry eye conditions, corneal cold nociception is further enhanced and leads to cold allodynia^22^. To investigate whether TRPV1 is involved in dry eye-induced cold allodynia, we generated a dry eye model in mice by surgically removing their exorbital lacrimal glands (intraorbital lacrimal glands remain intact)^23^. In this model, tear secretion was significantly reduced, and the epithelial erosions of the cornea were remarkably increased as revealed under a cobalt blue light after fluorescein staining (Fig. 5a–c). We found that *Trpv1*^*−/−*^ mice displayed normal basal tear secretion before the dry eye surgery. After surgery, *Trpv1*^*−/−*^ mice showed similar extents of reduced tear secretion and intensified corneal epithelial erosions as WT controls (Fig. 5b, c), indicating that TRPV1 is not involved in the pathogenesis of dry eye in this model.

After establishment of chronic dry eye (4 weeks after surgery), we found that the percentage of TRPM8^+^ neurons is not altered (Supplementary Fig. 7). However, TRPV1 is significantly upregulated in TRPM8^+^ cold-sensing neurons in dry eye mice, compared with sham-operated mice (Fig. 5d). Correlating well with this observation, more cold-sensitive neurons responded to capsaicin in the cornea-projection area of TG from dry eye mice (Fig. 5e), suggesting an enhanced nociceptive function of corneal TRPM8^+^ sensory fibers under dry eye conditions. Indeed, using normally innocuous doses of cryosim-3, activation of corneal TRPM8^+^ sensory fibers evoked nocifensive behavior in dry eye mice but not in sham-operated mice (Fig. 5f). Furthermore, dry eye mice displayed cold allodynia and showed eye closing response to air flow at 19 °C, which was not observed in sham-operated mice (Fig. 5g). These results confirmed an increased nociceptive function of corneal TRPM8^+^ sensory fibers under dry eye conditions, which was not found in previous study mainly due to discrepancy in methodologies (Supplementary Fig. 8)^24^. *Trpv1*^*−/−*^ mice with dry eye displayed significantly reduced eye closing responses to cryosim-3 and innocuous cold stimulus (19 °C), compared with WT controls (Fig. 5f, g). Furthermore, pharmacological blockade of TRPV1 using AMG9810 effectively alleviated dry eye-associated cold allodynia in WT mice (Fig. 5h). Together, these results indicate that TRPV1 is indispensable for dry eye-induced cold allodynia, and pharmacological suppression of TRPV1 is a promising therapeutic strategy for dry eye-associated cold allodynia.

### TRPV1 overexpression in TRPM8^+^ neurons causes cold allodynia

**Fig. 6:**
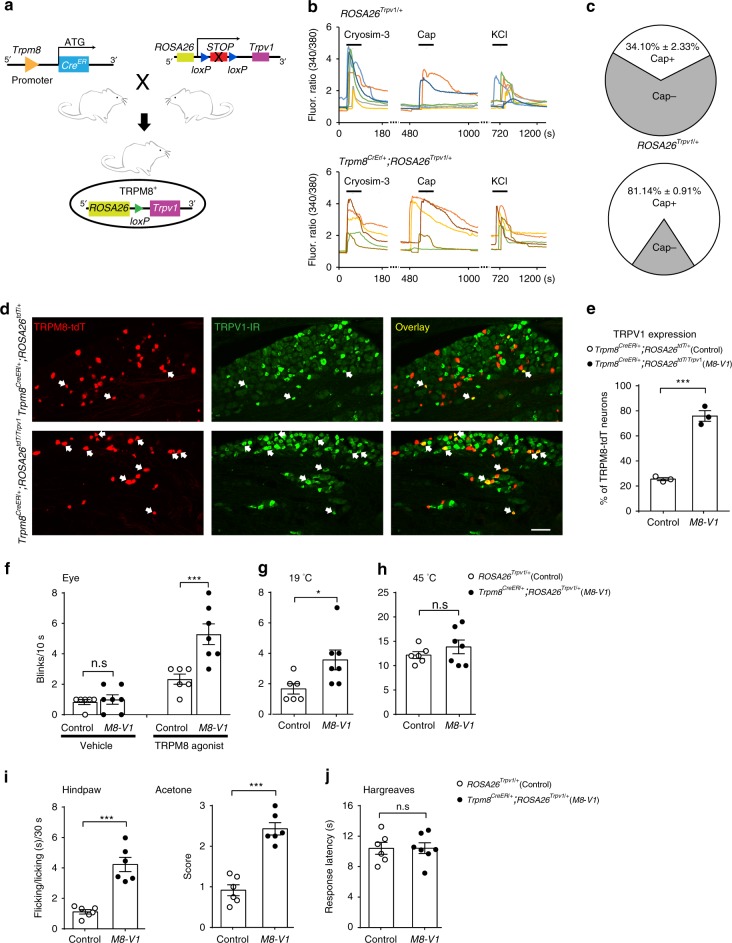
TRPV1 overexpression in TRPM8^+^ neurons results in cold allodynia. **a** Schematic diagram for the generation of *Trpm8*^*CreER/+*^*; ROSA26*^*Trpv1/+*^ mice. **b** Representative calcium transients of TRPM8^+^ neurons in response to the TRPM8 agonist cryosim-3 (10 µM) and TRPV1 agonist capsaicin (Cap, 1 µM) in *Trpm8*^*CreER/+*^*; ROSA26*^*Trpv1/+*^ mice and control *ROSA26*^*Trpv1/+*^ mice. **c** Quantification indicates that a significantly increased fraction of TRPM8^+^ neurons respond to capsaicin in *Trpm8*^*CreER/+*^*; ROSA26*^*Trpv1/+*^ mice (*n* = 5), compared with control *ROSA26*^*Trpv1/+*^ mice (*n* = 3). **d** Representative images showing TRPV1-immunoreactivity (TRPV1-IR, green) in tdTomato-labeled TRPM8^+^ neurons (Tdt, red) from *Trpm8*^*CreER/+*^; *ROSA26*^*Trpv1/tdTomato*^ and control *Trpm8*^*CreER/+*^; *ROSA26*^*tdTomato/+*^ mice. Arrows indicate TRPV1^+^/TRPM8^+^ neurons. **e** Quantification showing upregulated TRPV1-IR in TRPM8^+^ neurons from *Trpm8*^*CreER/+*^; *ROSA26*^*Trpv1/tdTomato*^ mice (*n* = 3), compared with control *Trpm8*^*CreER/+*^; ROSA26^*tdTomato/+*^ mice (*n* = 3). **f** TRPM8-agonist cryosim-3 (0.025 nmol in 1 μL) evoked significantly more reflex blinking in *Trpm8*^*CreER/+*^*; ROSA26*^*Trpv1/+*^ mice (*n* = 7) than in control *ROSA26*^*Trpv1/+*^ mice (*n* = 6). The vehicle (0.9% NaCl) did not induce significant reflex blinking in either *Trpm8*^*CreER/+*^*; ROSA26*^*Trpv1/+*^ (*n* = 7) or *ROSA26*^*Trpv1/+*^ mice (*n* = 6). **g** Innocuous cold (19 °C) evoked significantly more reflex blinking in *Trpm8*^*CreER/+*^*; ROSA26*^*Trpv1/+*^ mice (*n* = 7) than in control *ROSA26*^*Trpv1/+*^ mice (*n* = 6). **h** Noxious heat (45 °C) elicited similar ocular nociception-associated reflex blinking responses in both *Trpm8*^*CreER/+*^*; ROSA26*^*Trpv1/+*^ (*n* = 7) and control *ROSA26*^*Trpv1/+*^ mice (*n* = 6). **i** Evaporative cooling evoked by acetone applied onto the paw skin elicited more significant nociception-associated flicking and licking responses in *Trpm8*^*CreER/+*^*; ROSA26*^*Trpv1/+*^ mice (*n* = 6) than in control *ROSA26*^*Trpv1/+*^ mice (*n* = 6). **j** *Trpm8*^*CreER/+*^*; ROSA26*^*Trpv1/+*^ (*n* = 7) and control *ROSA26*^*Trpv1/+*^ mice (*n* = 6) display similar sensitivity to heat in the Hargreaves test. Data are expressed as mean ± s.e.m. Statistical analysis by two tailed Student’s *t*-test. n.s. not significant; *P < 0.05; ***P < 0.001. All images shown are representative of three independent experiments using tissues from at least three different mice. Scale bar: 100 μm. Source data are provided as a Source Data file.

Although our gene deletion and pharmacological blockade assays have shown that TRPV1 is necessary for dry eye-associated cold allodynia, it is unclear whether the upregulation of TRPV1 in TRPM8^+^ neurons alone (without dry eye) is sufficient for generating cold allodynia. To address this question, we generated *Trpm8*^*CreER/+*^; *ROSA26*^*Trpv1/+*^ mice in which TRPV1 is selectively overexpressed in TRPM8^+^ neurons (Fig. 6a). This line mimics the upregulation of TRPV1 in TRPM8^+^ neurons under pathological conditions without affecting the TRPV1 expression in TRPM8-negative neuronal populations. Moreover, this line circumvents dry eye-associated pathological changes and inflammation, which may introduce confounding factors in studying the involvement of TRPV1.

We first examined the expression of the *Trpv1* transgene in TRPM8^+^ neurons by conducting calcium imaging of primary sensory neurons. Of TRPM8^+^ TG neurons (identified by their responsiveness to cryosim-3) from control *ROSA26*^*Trpv1/+*^ mice, 34.10% ± 2.33% responded to capsaicin (Fig. 6b, c). In contrast, 81.14% ± 0.91% of TRPM8^+^ neurons from *Trpm8*^*CreER/+*^*; ROSA26*^*Trpv1/+*^ mice were sensitive to capsaicin, suggesting that the *Trpv1* transgene significantly increased TRPV1 expression in TRPM8^+^ neurons.

To confirm the overexpression of TRPV1 in TRPM8^+^ neurons from *Trpm8*^*CreER/+*^*; ROSA26*^*Trpv1/+*^ mice, we generated *Trpm8*^*CreER/+*^*; ROSA26*^*Trpv1/tdTomato*^ mice in which TRPM8^+^ neurons express both *Trpv1* and *tdTomato* transgenes. We found that significantly more tdTomato-labeled TRPM8^+^ neurons express TRPV1 in *Trpm8*^*CreER/+*^*; ROSA26*^*Trpv1/tdTomato*^ mice than in control *Trpm8*^*CreER/+*^*; ROSA26*^*tdTomato/+*^ mice which lack the *Trpv1* transgene (Fig. 6d, e), correlating well with our calcium imaging results.

We next examined the behavioral consequence of TRPV1 overexpression in TRPM8^+^ neurons. *Trpm8*^*CreER/+*^*; ROSA26*^*Trpv1/+*^ mice displayed significantly enhanced responses to both cryosim-3 and innocuous cold (19 °C), compared with littermate controls (Fig. 6f, g). This finding supports that TRPV1 upregulation in TRPM8^+^ neurons alone is sufficient for generating ocular cold allodynia. In contrast, the ocular response to heat was not altered in this transgenic mouse line (Fig. 6h), arguing against the effects of TRPV1 upregulation in TRPM8^+^ neurons on the thermal sensitivity of the cornea.

To determine whether the upregulation of TRPV1 is required for cold allodynia in tissues other than the cornea, we further examined whether the overexpression of TRPV1 results in similar cold allodynia in the skin. *Trpm8*^*CreER/+*^*; ROSA26*^*Trpv1/+*^ mice display cold allodynia in response to acetone applied to the hind paw skin (Fig. 6i), but an unaltered response to heat stimulation in the Hargreaves test (Fig. 6j). This finding indicates that the overexpression of TRPV1 in TRPM8^+^ neurons evokes cold allodynia, but does not change the thermal sensitivity of the skin. It provides a mechanism for skin cold allodynia under pathological conditions. Correlating well with the gain-of-function assay, TRPV1 deficiency results in less severe cold allodynia in the inflammatory pain model induced by complete Freund's adjuvant (Supplementary Fig. 9), substantiating an indispensable role of TRPV1 in cold allodynia. In summary, our results indicate that targeted overexpression of TRPV1 in TRPM8^+^ neurons is sufficient for generating cold allodynia in both the cornea and skin.

### TRPV1 facilitates substance P release and cold nociception

**Fig. 7:**
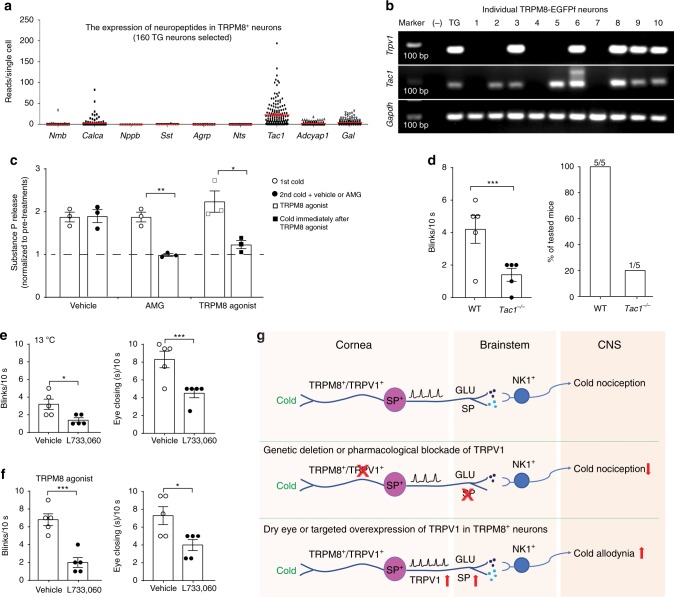
Substance P release is required for corneal cold nociception. **a** The expression of neuropeptides in mouse TRPM8^+^ trigeminal ganglionic (TG) neurons based on single-cell RNA-seq data. Each dot represents a single TRPM8^+^ neuron. Full dataset and methods are available in Nguyen et al.^25^. **b** Single-cell RT-PCR using intron-spanning primers was performed on individual TRPM8-EGFPf sensory neurons that project to the cornea. All TRPM8^+^/TRPV1^+^ neurons express substance P (*Tac1*), while fewer TRPM8^+^/ TRPV1^-^ neurons express *Tac1*. Negative control (−): No reverse transcription reaction on RNA sample from whole TG. Positive control (TG): cDNA from whole TG. **c** Cold treatments (bath temperature drops from 32 to15 °C) promote the release of substance P from dissociated trigeminal neurons, as revealed by ELISA assays. TRPV1 antagonist AMG9810 (AMG, 300 nM, 3 min pre-treatment before cold stimulations) suppressed the release of substance P, compared with the vehicle control (0.0006% DMSO). Cold-associated release of substance P was also suppressed by pre-desensitization of TRPM8^+^ neurons using TRPM8 agonist cryosim-3 (10 µM). **d** Both the reflex blinking and eye closing responses to air flow at 13 °C were significantly reduced in *Tac1*^*−/−*^ mice, compared with WT controls (*n* = 5 mice/group). **e**–**f** NK1 antagonist alleviates cold nociception. The reflex blinking and eye closing responses to cold (air flow at 13 °C) and cryosim-3 (0.1 nmol in 1 μL) were significantly reduced 30 min after intracisternal injection of NK1 antagonist L733,060 hydrochloride (10 µg in 5 µL, *n* = 5 mice), compared with the vehicle-treated group (0.9% NaCl, *n* = 5 mice). **g** Model for neural pathways encoding corneal cold nociception and allodynia. SP: substance P. Data are expressed as mean ± s.e.m. Statistical analysis by two tailed Student’s *t*-test. * *P* < 0.05, **P < 0.01, ***P < 0.001. Source data are provided as a Source Data file.

To understand the neural mechanism by which the co-expression of TRPV1 in TRPM8^+^ cold-sensing fibers promotes ocular cold nociception, we investigated the potential neurotransmitters/neuromodulators in signaling ocular cold nociception. We reanalyzed one previously published dataset of TG neuronal transcriptional profiles at the single cell level (GEO database, accession number GSE101984)^25^. In addition to vesicular glutamate transporter 2, substance P (encoded by *Tac1* gene) is unexpectedly highly expressed by a majority of TRPM8^+^ neurons in TG (Fig. 7a and Supplementary Fig. 10). To confirm the expression of substance P in corneal sensory neurons, we manually picked TG neurons that were retrogradely labeled by WGA-AF555 from the cornea. Single cell RT-PCR indicated that all TRPM8^+^; TRPV1^+^ neurons express substance P (*Tac1*), while fewer TRPM8^+^; TRPV1^-^ neurons express *Tac1* (Fig. 7b).

To determine whether substance P is a neurotransmitter for signaling ocular cold nociception, we examined the release of substance P after cold stimulation of dissociated sensory neurons (bath temperature drop from 32 °C to 15 °C). Indeed, cold induces a significant release of substance P (Fig. 7c). This release was suppressed by pre-desensitization of TRPM8^+^ neurons using cryosim-3 (Fig. 7c), substantiating the release of substance P from TRPM8^+^ neurons in response to cold. Studies have shown that higher frequencies of primary afferent stimulation are required to evoke the release of substance P, compared with that of glutamate^26,27^. As pharmacological blockade of TRPV1 using AMG9810 significantly reduces the depolarization and firing of TRPM8^+^ neurons, we examined whether similar treatments of AMG9810 reduces the release of substance P. Indeed, AMG9810 significantly reduced cold-induced substance P release from sensory neurons (Fig. 7c), suggesting that TRPV1 potentiates the release of substance P upon cold stimulation. To determine whether TRPV1 promotes cold nociception in a substance P-dependent manner, we examined whether substance P is required for signaling cold nociception. We found that *Tac1* deficiency significantly reduces eye closing as well as reflex blinking responses to cold (Fig. 7d), supporting that substance P is required for signaling ocular cold nociception.

We further examined whether substance P interacts with its postsynaptic receptor NK1 for synaptic transmission of cold nociceptive signal. Intracisternal injection of the NK-1 antagonist L733,060 hydrochloride into WT mice significantly reduced their reflex blinking and eye closing responses to cold and cryosim-3 (Fig. 7e, f), supporting that NK-1 is required for synaptic transmission of corneal TRPM8^+^ cold-sensitive neurons. Collectively, our results indicate that the expression of TRPV1 in TRPM8^+^ neurons facilitates cold-induced release of substance P, which interacts with postsynaptic NK1 receptors for signaling cold nociception (Fig. 7g).

## Discussion

Our results reveal a previously unrecognized function of TRPV1 in promoting ocular cold nociception. The expression of TRPV1 defines two major subsets of TRPM8^+^ sensory fibers in the mouse cornea (TRPV1^+^ *vs*. TRPV1^-^). We found that the TRPV1^+^ subset displays greater membrane depolarization and fires more action potentials than the TRPV1^-^ subset. This co-expression of TRPV1 and TRPM8 is required for cold nociception and allodynia by enhancing responsiveness of TRPM8^+^ neurons to cold and facilitating the release of neuropeptide substance P. Substance P is indispensable for the communication of TRPM8^+^ neurons with postsynaptic neurons for cold nociceptive signaling.

Under pathological dry eye conditions, the expression of TRPV1 is further upregulated in TRPM8^+^ neurons. This correlates well with a previous finding that TRPV1 protein levels were increased in anterior eye segment and trigeminal ganglion samples from rats with dry eyes, whereas TRPM8 levels were not affected^24^. The upregulation of TRPV1 in TRPM8^+^ neurons leads to severe cold allodynia, which can be effectively attenuated by genetic deletion or pharmacological blockade of TRPV1. Importantly, TRPV1 upregulation is not only necessary but also sufficient for generating cold allodynia. In the absence of pathological changes, targeted overexpression of TRPV1 in TRPM8^+^ sensory neurons using molecular genetics is sufficient for generating cold allodynia in both the corneal and skin.

The up-regulation of TRPV1 may represent a general mechanism that enhances excitability of TRPM8^+^ cold-sensing neurons and other sensory neurons. Studies have shown that TRPV1 displays tonic activity at body temperature^13,14^. The up-regulation of TRPV1 may lead to spontaneous activity in corneal TRPM8^+^ neurons. Indeed, studies have shown that the cold-sensitive fibers in the cornea display the ongoing impulse activity at 34 °C^2^. The expression of TRPV1 may be essential to this ongoing activity in TRPM8^+^ sensory fibers and enable TRPM8^+^ neurons to depolarize and fire action potentials more readily, the latter of which has been supported by our electrophysiological recording results in Fig. 3.

However, this enhanced neuronal excitability mediated by TRPV1 does not alter the thermal sensitivity of mice, based on ours and others' studies^26,27^. Previous studies have shown that ablation of TRPM8^+^ neurons leads to cold insensitivity, but does not alter the thermal sensitivity^28,29^, suggesting that TRPM8^+^ neurons are specific for cold perception and are not required for thermal sensitivity. Hence TRPV1 expressed in TRPM8^+^ neurons is not necessary for thermal sensitivity. Our results further show that targeted overexpression of TRPV1 in TRPM8^+^ neurons does not cause altered thermal responses in both the cornea and skin (Fig. 6), substantiating the dispensable role of TRPV1 in TRPM8^+^ neurons in regulating thermal sensitivity.

The unexpected role of TRPV1 in corneal cold-sensing fibers suggests its potential as a therapeutic target for ocular cold allodynia. The high co-expression of TRPV1 and TRPM8 in primary afferent fibers is unique to the cornea, but not other tissues examined, revealing a mechanism underlying the tissue difference in nociceptive sensitivity to cold. Of note, although TRPM8 is required for ocular cold nociception, it is not a good drug target for ocular pain management, as it regulates basal tearing and maintains the ocular surface wetness^2^. Antagonizing TRPM8 for alleviating dry eye-associated cold allodynia can reduce basal tearing and aggravate dry eye symptoms. In contrast, TRPV1, as a nociceptive channel, is a more promising drug target than TRPM8 for treating cold allodynia. Our previous studies have shown that antagonizing TRPV1 could effectively block histamine-dependent itch in allergic conjunctivitis^30^, suggesting a potentially wide use of TRPV1 antagonists in alleviating ocular discomforts associated with different ocular conditions. Furthermore, the activation of TRPM8^+^/TRPV1^+^ neurons by cold causes the release of substance P, which has been shown to mediate neurogenic inflammation^31,32^. Pharmacological blockade of TRPV1 decreases substance P release (Fig. 7c), and hence would attenuate associated inflammation and disease progression. Finally, the upregulation of TRPV1 in TRPM8^+^ neurons is sufficient to generate cold allodynia in both corneal and non-corneal tissues, which offers a general mechanism underlying cold allodynia associated with different conditions in different tissues. We have shown that TRPV1 is required for cold allodynia under inflammatory pain conditions in the skin (Supplementary Fig. 9). It would be interesting to further study whether TRPV1 is involved in cold allodynia associated with sensitive teeth and fibromyalgia affecting bones and muscles. In summary, our study sheds light on unexpected functions of TRPV1 in cold nociception and provides a common mechanism behind the corneal cold nociception and pathological cold allodynia, which opens an avenue for pain management.

## Methods

### Animals

C57BL/6J wild-type (WT, Stock#: 000664), *Trpm8*^*−/−*^ (Stock#: 008198), *Trpv1*^*−/*−^ (Stock#: 003770), *ROSA26*^*Trpv1/+*^ (Stock#: 008513), *Tac1*^−*/*−^ (Stock#: 004103) and *ROSA26*^*tdTomato/+*^ (Stock#: 007905) mice were ordered from Jackson Laboratory. *Pirt*^*GCaMP3/+*^ mice^18^ were gifted by Dr. Xinzhong Dong at Johns Hopkins University. *Trpm8*^*EGFPf/+*^ mice^10^ were gifted by Dr. Gina Story when she worked at Washington University Pain Center. *Trpm8*^*creER/+*^ mice were from Dr. Hongzhen Hu at Washington University Center for the Study of Itch and Sensory Disorders. All transgenic animals used for behavioral experiments were backcrossed to the C57BL/6J background, and all mice were between two and three months old at the time of experiment. Researchers were blinded to mouse genotype throughout experiment and analysis. All experimental procedures were approved by the Institutional Animal Care and Use Committee at Washington University in St. Louis, School of Medicine, and complied with all relevant ethical regulations for animal testing and research.

### Cold and heat stimulations of the cornea

Cold and heat stimulations were applied using temperature-controlled air flow. Briefly, gentle air flow (0.5 L/min) passed through a PVC tube immersed into water bath, where the temperature was adjusted until the outflow air is maintained stably at the desired experimental temperatures. During testing, mice were restrained manually and their eyes were placed 5 mm away from the air flow. Blinking and eye closing responses were videoed for 10 s. Blinking is rapid, forceful eye closing followed by immediate eye opening. In contrast, eye closing reflects significant ocular width/length ratio changes over time and is not followed by immediate eye opening. In our study, mice display obvious eye closing when their ocular width/length ratio drops by 50% or more from their basal ratio. The ocular width/length ratio was calculated based on the measurement of the width and length of the palpebral fissure^16^.

### Chemical application to the cornea

After acclimation, test animals were manually restrained and test compounds (e.g. capsaicin, cryosim-3, menthol, isotonic and hypertonic saline) were carefully applied onto the cornea using a pipette. Blinking and eye closing responses were scored for 10 s immediately after chemical application.

### Retrograde labeling

Adult mice were anesthetized with ketamine (100 mg/kg) and xylazine (10 mg/kg) cocktail and placed under a stereoscopic dissection microscope. Approximately 0.5 μL of 4% fluorogold (Cat# 80014, Biotium) or 5 mg/mL WGA-Alexa Fluor® 555 (W32464, Life Tech) in PBS was injected into the cornea with a pulled glass micropipette. Mice were euthanized for immunohistochemistry staining, single cell PCR or whole trigeminal ganglia calcium imaging three days after dye injection.

### Immunofluorescence staining

Mice were perfused with ice cold PBS (pH 7.4) followed by 4% paraformaldehyde (PFA) in PBS after CO_2_ euthanasia. Trigeminal ganglia (TG) and brainstems were dissected and post-fixed in 4% PFA at 4 °C for 30 min and 4 h, respectively. Tissues were cryoprotected in 30% (w/v) sucrose overnight at 4 °C and frozen in OCT for sectioning. TG was sectioned at 12 μm onto slides and brainstem was sectioned at 30 μm for floating staining. Immunofluorescence staining was done as in a previous study^33^. Briefly, sectioned tissues were blocked with 10% goat serum in PBST (PBS containing 0.1% Triton X-100) for 1 h and incubated with primary antibodies at 4 °C overnight. After rinsing, sections were incubated with secondary antibody for 2 h at room temperature. Images were taken and analyzed using Nikon fluorescence microscope with a CoolSnap HQ2 CCD camera (Photometrics, Tucson, AZ).

Primary antibodies used: rabbit anti-TRPV1 (VR1-C; RA14113; Neuromics; 1:1000), chicken anti-GFP (GFP-1020; Aves Lab; 1:1000).

Secondary antibodies used: goat anti-rabbit IgG (Alexa Fluor-555; A21429, Life technologies; 1:500), donkey anti-chicken IgG (114050, FITC conjugated; Jackson ImmunoResearch; 1:500).

### Calcium imaging

KNRK cells were co-transfected with TRPM8 and TRPV1 plasmids^34,35^. KNRK cells were derived from normal rat kidney transformed by Kirsten sarcoma virus (ATCC® CRL-1569™), and have been widely used for heterologous expression of G-protein couple receptors and channels^36–41^.

Transfected KNRK cells or dissociated TG neurons from *Trpm8*^*CreER/+*^*; ROSA26*^*Trpv1/+*^ mice were loaded with Fura-2 (Molecular Probes, F1221) in calcium imaging buffer (CIB, containing (in mM): NaCl 130, MgCl_2_ 0.6, KCl 3, NaHCO_3_ 1.2, CaCl_2_ 2.5, HEPES 10, and glucose 10, pH 7.40) for 40 min and recovered for 10 min before assay. Whole-mount TG from *Pirt*^*GCaMP3/+*^ mice were dissected and recovered for 60–90 min at RT in freshly prepared synthetic interstitial buffer (SIF, containing (in mM): NaCl 107.8, MgSO_4_ 0.69, KCl 3.5, CaCl_2_ 1.53, NaH_2_PO_4_ 1.67, NaHCO_3_ 26.2, C_6_H_11_NaO_7_ 9.64, glucose 5.55, sucrose 7.6, bubbled with 5% CO_2_ balanced O_2_ to yield pH 7.40 before use). The temperature of bath (CIB for cultured cells and SIF for whole TG) was controlled by a dual in-line heater/cooler regulator (Warner Instruments, SC-20). The cold stimulation was generated by bath temperature drop from 32 °C to 15 °C. Compounds including capsaicin (Sigma, M2028), AMG9810 (Tocris, 2316), and KCl were bath applied. AMG9810 was dissolved in DMSO as stock solution and diluted to 300 nM with CIB or SIF immediately before use. The pretreatment of AMG9810 or vehicle (diluted DMSO in CIB) was 3 min for transfected KNRK cells, and 10 min for sensory neurons before cold stimulation. Cellular response was acquired using an inverted Nikon fluorescence microscope with a CoolSnap HQ_2_ CCD camera (Photometrics) and quantified offline with the Nikon-NIS program. A minimal 15% increase of ΔF/F_0_ in 340/380 ratio or GCaMP3 fluorescence intensity were used as threshold response.

### Electrophysiology

Dissociated TRPM8^+^ TG neurons from *Trpm8*^*EGFPf/+*^ mice were first screened for capsaicin sensitivity using calcium imaging. Whole-cell current-clamp recordings were performed using a MultiClamp 700B amplifier and pCLAMP 10.5 software (Axon Instruments). Internal solution contained (in mM): K^+^-gluconate 120, KCl 30, MgCl_2_ 2, HEPES 10, MgATP 2, CaCl_2_ 1, EGTA 11, with pH adjusted to 7.2 using Tris-base. Neurons were continuously perfused (2–3 mL/min) with external solution containing (in mM): NaCl 145, KCl 3, CaCl_2_ 2, MgCl_2_ 2, glucose 10 and HEPES 10, pH 7.4. Solution temperature was maintained at 33 °C using a dual in-line heater/cooler regulator (Model SC-20, Warner Instruments, Hamden, CT 06514).

Resting membrane potential (RMP) was recorded after stabilization (within 4 min) and unhealthy neurons (with RMP > −45 mV) were excluded from the study. Cold stimulation was introduced by lowering the bath temperature to 10 °C. Neurons were allowed to recover for ~10 min at 33 °C, before bath perfusion of AMG9810 or vehicle containing external solution.

### Mouse dry eye model

Adult mice were anesthetized using a ketamine (100 mg/kg) and xyaline (10 mg/kg) cocktail and 5 mm incisions were made in the skin between the eye and the ear. The skin was carefully retraced and the exorbital lacrimal gland was gently isolated and removed^23^. Skin was sutured with 6–0 black monofilament nylon (ETHILON, INC.) and the procedure was repeated on the contralateral side. Sham-operated mice received the same procedure without gland removal.

All mice received antibiotics and analgesia for 2 days postoperatively. Corneal abrasion was evaluated under a cobalt blue light after application of 0.5 μL of 0.25% fluorescein sodium (Bausch & Lomb Inc. Tampa, FL 33637), and was scored based on area of corneal staining^42^. Tear volume was measured without anesthesia with phenol-red cotton threads (Zone-Quick; Showa Yakuhin Kako CO., Ltd, Tokyo, Japan)^43^. Briefly, the threads were held with forceps and applied gently to the lateral canthus for 30 s. Wetting of the threads was measured in millimeters under a dissection microscope.

### Tamoxifen administration

To induce CreER nuclear translocation in *Trpm8*^*creER/+*^ mice, animals were administered tamoxifen (T5648, Sigma) by oral gavage, according to a protocol adapted from a previous study^44^. In brief, tamoxifen was freshly prepared daily at 20 mg/mL in olive oil (O1514, Sigma) and administered for six consecutive days, starting at P28. Dose administered was 40 mg/kg of body weight per day. Mice were used for experiments 4 weeks after the completion of the tamoxifen regimen.

### Single cell RT-PCR

To isolate corneal TRPM8^+^ neurons, TG from *Trpm8*^*EGFPf/+*^ mice with corneal WGA-AF555 injections were dissected and dissociated. TG neurons were purified using a 15% BSA density gradient column. EGFPf^+^/AF555^+^ neurons cells were visually identified under a Leica DMi6000 microscope (Buffalo Grove, IL) and manually picked using a Narishige MMO-202ND micromanipulator (Amityville, NY). Isolated TG neurons were ejected into PCR tubes containing 10 µL of lysis buffer and RNase inhibitor (18080200, Life Tech), and flash frozen on dry ice and stored at −80 °C until cDNA synthesis.

cDNA was generated using Invitrogen SuperScript III CellsDirect cDNA Synthesis Kit (18080200, Life Tech), according to the manufacturer’s recommended protocol. RT-PCR was performed using 2 µL cDNA and Qiagen HotStar Taq Polymerase (203203, Qiagen). RT negative control: No reverse transcription reaction on RNA sample from whole TG (to exclude the contamination from genomic DNA). Positive control: cDNA from the whole TG. Gene specific primers are listed in Table 1 . Uncropped images of the verified gels are provided in the Data Source.

**Table 1:** Genetic specific primers for single cell RT-PCR.

Gene	Forward Primer	Reverse Primer	Anneal	Amplicon Size (bp)
*Trpv1*	ACCACGGCTGCTTACTATCG	CGGAAATAGTCCCCAACGGT	52 °C	77
*Tac1*	CGGCCAAGGAGAGCAAAGA	ACGGCCACGAGGATTTTCAT	62 °C	96
*Gapdh*	CCCAGCAAGGACACTGAGCAA	TTATGGGGGTCTGGGATGGAAA	65 °C	93

### Enzyme-Linked Immunosorbent Assay (ELISA)

To measure substance P (SP) release, dissociated TG neurons were seeded onto the 7 mm glass window of a 35 mm glass bottom dish (MatTek Corporation) and cultured at 37 °C for 18–24 h before use. To stimulate the neurons, culture media was gently rinsed away, replaced with 50 µL of CIB containing vehicle or stimulants, and incubated at 37 °C for 5 min. Cold stimulation was generated by bath temperature drop from 32 °C to 15 °C. The interval between first and second cold stimulations was 5 h. SP containing supernatant was collected for Enzyme-Linked Immunosorbent Assay (ELISA).

SP concentration was measured using an ELISA kit (ADI-900–018, Enzo Life Sciences) and BioTek Synergy H4 plate reader. SP concentration was determined using the manufacturer’s supplied standards and recommended protocol.

### Intracisternal injection

To pharmacologically block the substance P receptor, mice received intracisternal injection of NK1-angtagonist L733,060 hydrochloride. Briefly, adult C57BL/6 J wild-type male mice were anesthetized with 2% isoflurane during the procedure. L733,060 hydrochloride (10 µg in 5 µL) was injected slowly into the cisterna magna. Behavioral tests were performed 30 min after injection.

### Acetone test

Mice were placed in acrylic testing chambers on an elevated wire mesh floor. After a habituation period of 30 min, 15–20 µL of acetone was gently applied onto the plantar surface of the hind paw. Responses were scored and analyzed as previously described^45^, and were graded as follows: 0, no response; 1, brisk withdrawal or flick of the paw; 2, repeated flicking of the paw; 3, repeated flicking and licking of the paw^46^.

### Hargreaves test

Hargreaves test was used to measure the thermal sensitivity as previously described^47^. In brief, mice were placed in acrylic testing chambers on a heated (30 °C) glass plate (IITC Life Science). The radiant heat source was delivered to the plantar surface of the hind paw and withdrawal latencies were scored.

### Data analysis

Data are shown as means ± s.e.m. Statistical significances were determined using *t*-test (for two groups) or one-way ANOVA followed by a Tukey-Kramer post hoc test (for three or more groups). Difference between groups is considered statistically significant if *P* < 0.05.

### Reporting summary

Further information on research design is available in the Nature Research Reporting Summary linked to this article.

## Supplementary information

Supplementary Information

Reporting Summary

## Data Availability

The authors declare that the data supporting the findings of this study are available within the paper and its supplementary information files. The raw data underlying Figs. 1b, c, e, f, 2b, d, 3b, d, f, g, i, j, l, m, 4a–d, f, 5b–h, 6c, e–j, 7b–f and Supplementary Figs. 1, 2, 3, 4, 5, 6g, 7, 8, 9 and 10b are provided as a Source Data file. No restrictions on data availability.

## Source Data

Source Data
