# Supplementary material for: TRPV1 activity and substance P release are required for corneal cold nociception
Source: Nat Commun. 2019 Dec 12;10:5678. doi: 10.1038/s41467-019-13536-0 (PMC6908618; doi:10.1038/s41467-019-13536-0)
Supplement: Supplementary file 1 — Supplementary Information [file 41467_2019_13536_MOESM1_ESM.pdf]

TRPV1 activity and substance P release are required for corneal cold nociception

Li; Yang; Jiang et al.

Supplementary Figures

Supplementary Figure 1: The temperature changes of the corneal surface.
Supplementary Figure 2: Capsaicin elicits reflex blinking.

Supplementary Figure 3: TRPA1 channel is not required for corneal cold nociception.

Supplementary Figure 4: The ocular width/length ratio changes of WT and *Trpm8*$^{-/-}$ mice in response to cold.

Supplementary Figure 5: A water-soluble TRPM8-specific agonist cryosim-3 elicits reflex blinking.

Supplementary Figure 6: TRPV1 antagonist does not have off-target effects on TRPM8.

Supplementary Figure 7: Proportion of corneal TRPM8-neuron is not increased in dry-eye mice.

Supplementary Figure 8: Menthol and hypertonic NaCl solution induce reflex blinking and eye closing responses in WT mice

Supplementary Figure 9: TRPV1 deficiency results in attenuated cold allodynia in the inflammatory pain model induced by complete freund's adjuvant (CFA).

Supplementary Figure 10: Mouse corneal TRPM8^{+} sensory neurons express vesicular glutamate transporter 2 (*Vglut2*).

Supplementary Figures

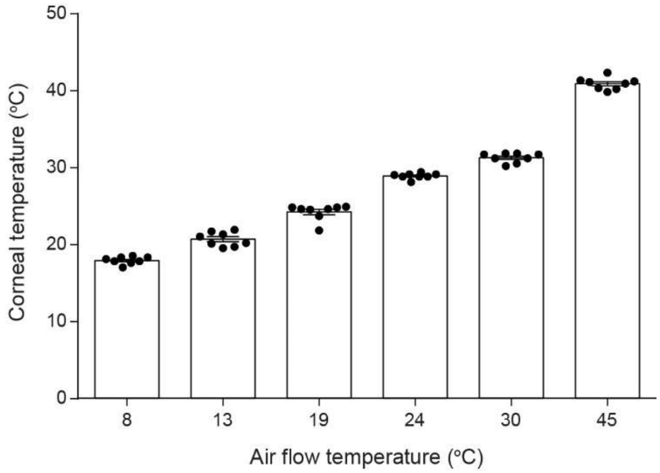

Supplementary Figure 1: The temperature changes of the corneal surface produced by the air flow at different temperatures applied to the ocular surface (n=8 WT mice / group). Data are expressed as mean ± s.e.m. Source data are provided as a Source Data file.

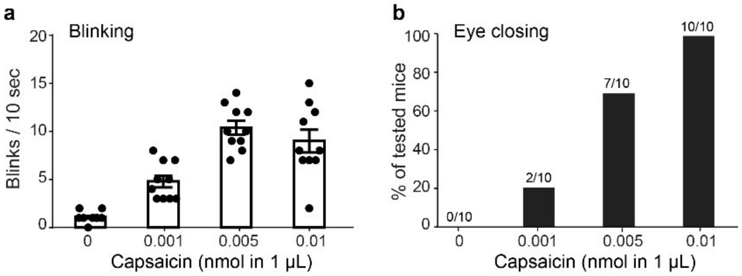

Supplementary Figure 2: Capsaicin elicits reflex blinking (**a**) and eye closing (**b**) in a dose-dependent manner in WT mice (n=10 / group). Data in **a** are expressed as mean±s.e.m. Source data are provided as a Source Data file.

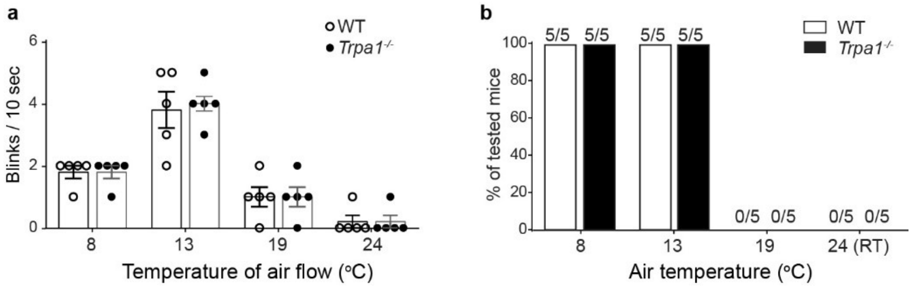

Supplementary Figure 3: TRPA1 channel is not required for corneal cold nociception. *Trpa1*$^{-/-}$ and WT mice display indistinguishable reflex blinking (**a**) and eye closing responses (**b**) to cold stimulations by the air flow at different temperatures (n=5 mice / genotype). Data are expressed as mean ± s.e.m. Source data are provided as a Source Data file.

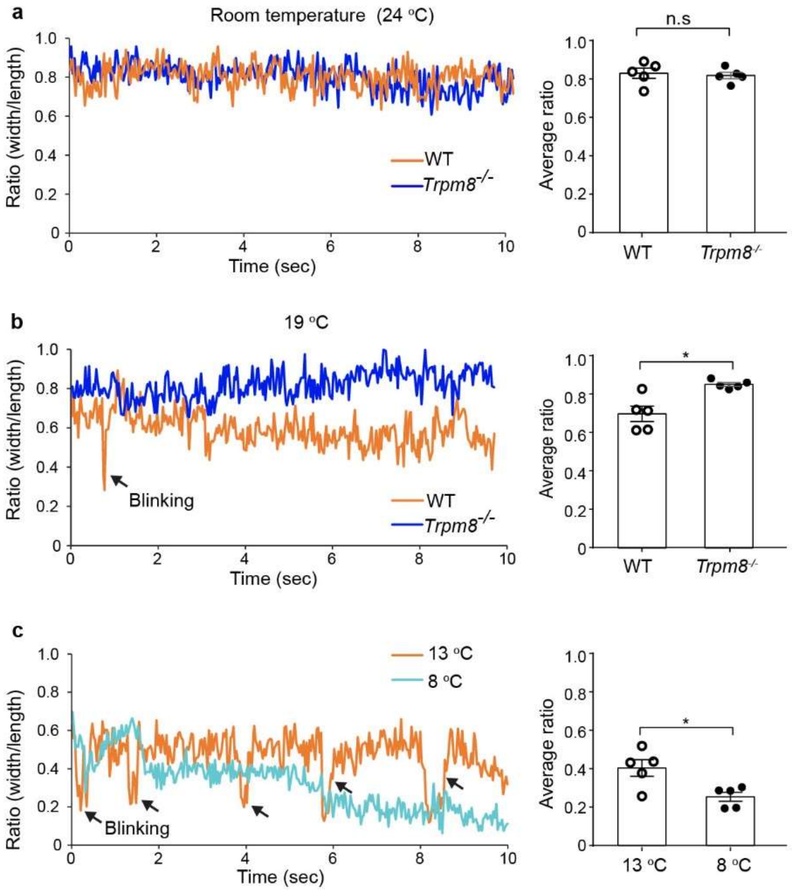

Supplementary Figure 4: The ocular width/length ratio changes of WT and *Trpm8*$^{-/-}$ mice in response to cold. (**a**) The representative width/length ratio curves and group analysis reveal no significant difference between WT (n=5) and *Trpm8*$^{-/-}$ mice (n=5) in their basal ratio at room temperature. (**b**) The representative "width/length" ratio curves and group analysis reveal a significant difference between WT (n=5) and *Trpm8*$^{-/-}$ mice (n=5) in response to the air flow at 19 °C. (**c**) The representative width/length ratio curves show that WT mouse closed its eyes upon exposure to the air flow at 8 °C and blinked fewer times than at 13 °C. Arrows indicate blinking responses to 13°C. Group analysis indicates that the average width/length ratio of WT mice in response to the cold stimulation of 8°C is significantly lower than to 13°C (n=5 / group). Data are expressed as mean ± s.e.m. Statistical analysis by two tailed Student's t-test. n.s. not significant; * $P < 0.05$. Source data are provided as a Source Data file.

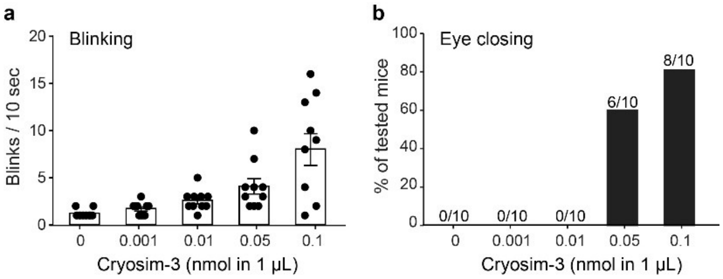

Supplementary Figure 5: A water-soluble TRPM8-specific agonist cryosim-3 elicits reflex blinking (**a**) and eye closing (**b**) in a dose-dependent manner in WT mice (n=10 / group). Notably, eye closing was evoked only by high doses of cryosim-3, suggesting that eye closing is indicative of corneal nociception to intense chemical stimuli. Data in **a** are expressed as mean±s.e.m. Source data are provided as a Source Data file.

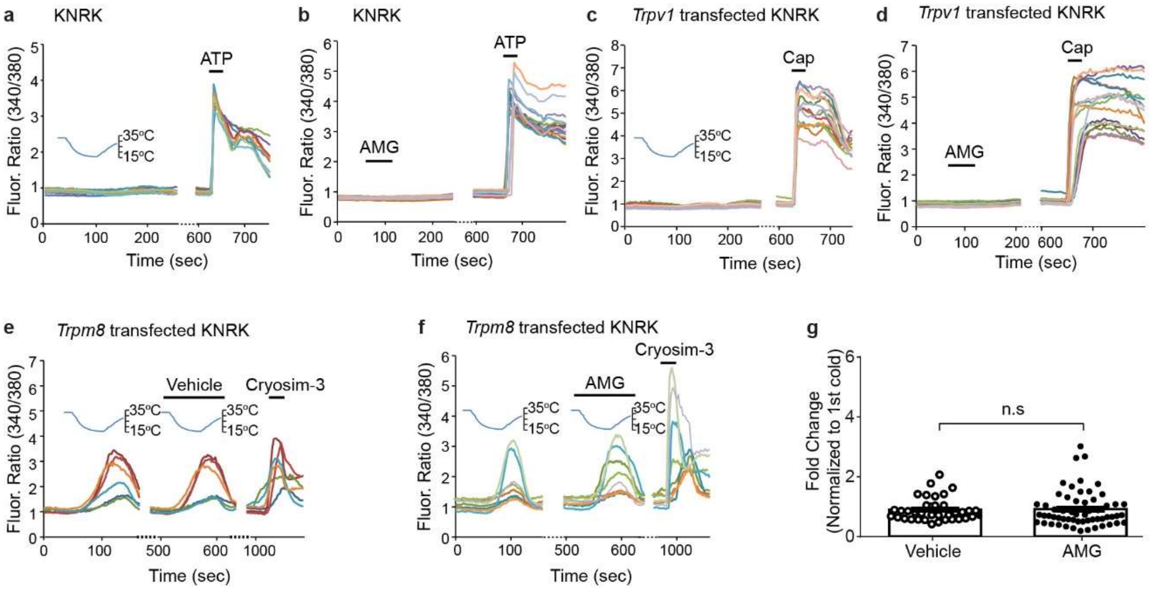

Supplementary Figure 6: TRPV1 antagonist (AMG9810) does not have off-target effects on TRPM8. (**a-d**) Representative calcium transients of naïve or *Trpv1*-transfected KNRK cells in response to cold or AMG9810. ATP (1 mM) and capsaicin (1 µM) serve as positive controls for naïve and *Trpv1*-transfected KNRK cells, respectively. (**e-f**) Representative calcium transients of *Trpm8*-transfected KNRK cells in response to cold in the absence or presence of vehicle or AMG9810 (300 nM). TRPM8 agonist cryosim-3 (10 µM) serves as a positive control for *Trpm8*-transfected KNRK cells. (**g**) Quantification of calcium responses of *Trpm8*-transfected cells in response to cold. Each dot represents one *Trpm8*-transfected KNRK cell. Data are expressed as mean ± s.e.m. Statistical analysis by two tailed Student's t-test. n.s. no statistical significance. Source data are provided as a Source Data file.

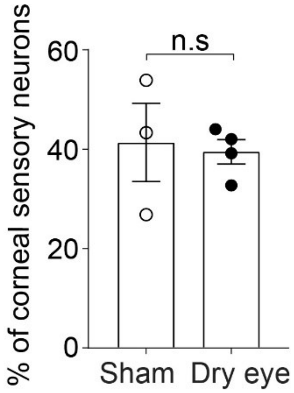

Supplementary Figure 7: The proportion of fluorogold-retrogradely-labeled corneal sensory neurons that express TRPM8-EGFPf is not increased in dry-eye mice (n=4), compared with control mice (n=3). Data are expressed as mean±s.e.m. No significant difference was found between control and dry eye groups by two tailed Student's t-test. n.s. no statistical significance. Source data are provided as a Source Data file.

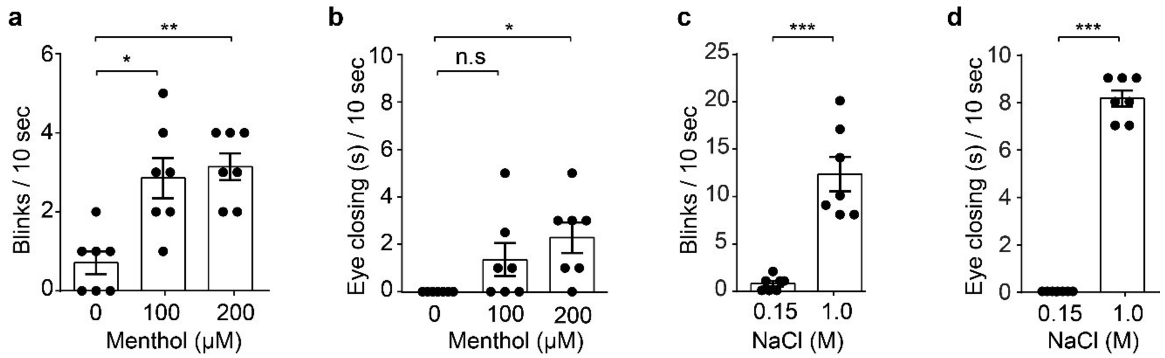

Supplementary Figure 8: Menthol and hypertonic NaCl solution induce reflex blinking and eye closing responses in WT mice**.** (**a-b**) Menthol solution induced significant reflex blinking and eye closing responses. (**c-d**) Hypertonic NaCl solution (1.0 M, 5.8% w/v) induced significantly more blinking and eye closing responses than isotonic NaCl solution (0.15 M, 0.9% w/v). n=7 mice / group. Data are expressed as mean ± s.e.m. * $P < 0.05$, ** $P < 0.01$, *** $P < 0.001$, n.s. not significant. Source data are provided as a Source Data file.

Note: A previous study has shown that eye wiping behavior was enhanced in dry eye rats after capsaicin instillation, but not after menthol when compared to sham rats[1]. Eye wiping is a nocifensive behavioral response to intense stimulation of eyes (e.g. capsaicin), while reflex blinking and eye closing are nocifensive behavior that can be evoked by less intense stimuli. Indeed, we found that hypertonic NaCl solution (1M with an osmolarity of 2000 mOsm/L) and menthol (100 and 200 μM) induce significant blinking and eye closing responses in mice (Supplementary Fig. 8), even though they failed to induce significant wiping behavior in sham or dry eye rats [1]. Due to discrepancy in methodologies, the previous study did not find the nociceptive function of corneal TRPM8$^+$ sensory fibers under dry eye conditions. However, we found that both low doses of cryosim-3 and normally innocuous cold induce reflex blinking and eye closing in dry eye mice (Fig. 5f), suggesting that TRPM8 is required for dry eye-associated cold allodynia.

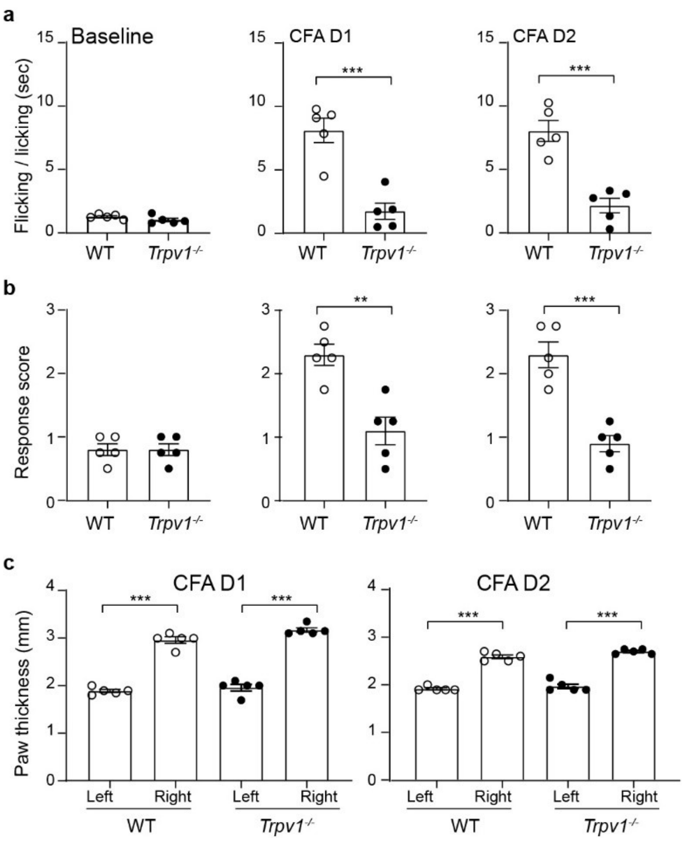

Supplementary Figure 9: TRPV1 deficiency results in attenuated cold allodynia in the inflammatory pain model induced by complete freund's adjuvant (CFA). (**a-b**) The flicking/licking duration and behavioral response score in the acetone evaporative test was significantly increased in the first two days after CFA injection (50%, 6 µL, mouse right paw) in WT mice (n=5), but not in *Trpv1*$^{-/-}$ mice (n=5). (**c**) CFA induced similar edema and swelling of the right paw (reflected as an increased paw thickness) in WT (n=5) and *Trpv1*$^{-/-}$ mice (n=5). The control left paw did not show edema or swelling. Each dot represents a mouse paw. Data are expressed as mean±s.e.m. Statistical analysis by two tailed Student's t-test. ***P*<0.01, ****P*<0.001. Source data are provided as a Source Data file.

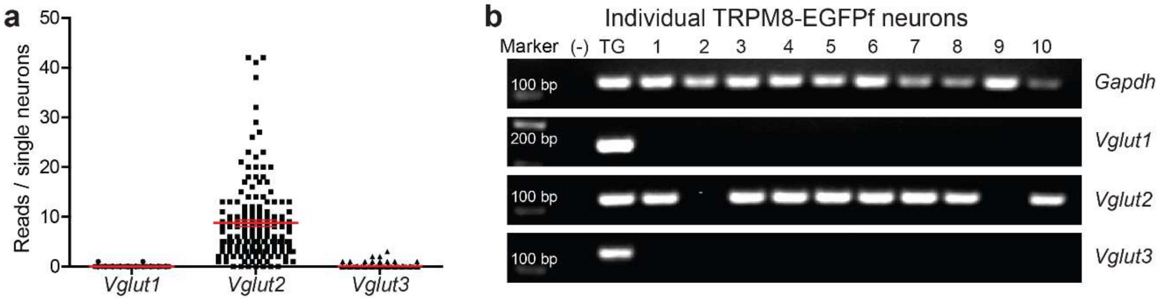

Supplementary Figure 10: Mouse corneal TRPM8$^+$ sensory neurons express vesicular glutamate transporter 2 (*Vglut2*). (**a**) The expression of vesicular glutamate transporters in mouse TRPM8$^+$ trigeminal ganglionic (TG) neurons based on single-cell RNA-seq data. Each dot represents a single TRPM8$^+$ neuron. Full dataset and methods are available in Nguyen et al [2]. (**b**) Single-cell RT-PCR using intro-spanning primers was performed on individual TRPM8$^{EGFPf/+}$ sensory neurons that project to the cornea. A majority of TRPM8$^{EGFPf/+}$ neurons express vesicular glutamate transporter 2 (*Vglut2*), but not vesicular glutamate transporter 1 or 3 (*Vglut1* or *Vglut3*). Negative control (-): No reverse transcription reaction on RNA sample from whole TG. Positive control (TG): cDNA from whole TG. Source data are provided as a Source Data file.

Supplementary References

1. Bereiter DA, Rahman M, Thompson R, Stephenson P, Saito H. TRPV1 and TRPM8 Channels and Nocifensive Behavior in a Rat Model for Dry Eye. *Invest Ophthalmol Vis Sci* **59**, 3739-3746 (2018).

2. Nguyen MQ, Wu Y, Bonilla LS, von Buchholtz LJ, Ryba NJP. Diversity amongst trigeminal neurons revealed by high throughput single cell sequencing. *PLoS One* **12**, e0185543 (2017).
