# Supplementary material for: TRPV1 activity and substance P release are required for corneal cold nociception
Source: Nat Commun. 2019 Dec 12;10:5678. doi: 10.1038/s41467-019-13536-0 (PMC6908618; doi:10.1038/s41467-019-13536-0)
Supplement: Supplementary file 2 — Reporting Summary [file 41467_2019_13536_MOESM2_ESM.pdf]

nature research

Corresponding author(s): Qin Liu

Last updated by author(s): Oct 9, 2019

Reporting Summary

Nature Research wishes to improve the reproducibility of the work that we publish. This form provides structure for consistency and transparency in reporting. For further information on Nature Research policies, see Authors & Referees and the Editorial Policy Checklist.

Statistics

For all statistical analyses, confirm that the following items are present in the figure legend, table legend, main text, or Methods section.

n/a	Confirmed	
☐	☒	The exact sample size (*n*) for each experimental group/condition, given as a discrete number and unit of measurement
☐	☒	A statement on whether measurements were taken from distinct samples or whether the same sample was measured repeatedly
☐	☒	The statistical test(s) used AND whether they are one- or two-sided<br>*Only common tests should be described solely by name; describe more complex techniques in the Methods section.*
☐	☒	A description of all covariates tested
☐	☒	A description of any assumptions or corrections, such as tests of normality and adjustment for multiple comparisons
☐	☒	A full description of the statistical parameters including central tendency (e.g. means) or other basic estimates (e.g. regression coefficient) AND variation (e.g. standard deviation) or associated estimates of uncertainty (e.g. confidence intervals)
☒	☐	For null hypothesis testing, the test statistic (e.g. *F*, *t*, *r*) with confidence intervals, effect sizes, degrees of freedom and *P* value noted<br>*Give P values as exact values whenever suitable.*
☒	☐	For Bayesian analysis, information on the choice of priors and Markov chain Monte Carlo settings
☒	☐	For hierarchical and complex designs, identification of the appropriate level for tests and full reporting of outcomes
☒	☐	Estimates of effect sizes (e.g. Cohen's *d*, Pearson's *r*), indicating how they were calculated

Our web collection on statistics for biologists contains articles on many of the points above.

Software and code

Policy information about availability of computer code

Data collection	Nikon NIS Elements AR software for calcium imaging data collection<br>pCLAMP 10.5 software (Axon Instruments, U.S.) and Patchmaster software (v2; Heka Electronic) for electrophysiology data collection
Data analysis	Nikon NIS Elements AR software for calcium imaging data analysis<br>pCLAMP 10.5 software (Axon Instruments, U.S.) and Patchmaster software (v2; Heka Electronic) for electrophysiology data analysis<br>GraphPad Prism5 for statistical analysis

For manuscripts utilizing custom algorithms or software that are central to the research but not yet described in published literature, software must be made available to editors/reviewers. We strongly encourage code deposition in a community repository (e.g. GitHub). See the Nature Research guidelines for submitting code & software for further information.

Data

Policy information about availability of data

All manuscripts must include a data availability statement. This statement should provide the following information, where applicable:

- Accession codes, unique identifiers, or web links for publicly available datasets
- A list of figures that have associated raw data
- A description of any restrictions on data availability

All data generated or analysed during this study are included in this published article (and its supplementary information files).

Field-specific reporting

Please select the one below that is the best fit for your research. If you are not sure, read the appropriate sections before making your selection.

☒ Life sciences ☐ Behavioural & social sciences ☐ Ecological, evolutionary & environmental sciences

For a reference copy of the document with all sections, see nature.com/documents/nr-reporting-summary-flat.pdf

Life sciences study design

All studies must disclose on these points even when the disclosure is negative.

Sample size	The criteria for determining the number of animals used in behavioral assays is based on the "Sample size determination" (Dell et al. 2002, ILAR J, 43(4), 207–13). Three factors are important to calculate sample size (Dell et al, 2002).<br>1. the size of the effect under study (difference between experimental groups)<br>2. the desired power of the experiment to detect the effect (usually 80-90%)<br>3. the significance level (we chose 0 .05).<br>The animals used in our current study were all age-matched, congenic C57BL/6 inbred male mice. Animals with the same genotype are genetically identical, while the only genetic differences between genotypes are at the indicated alleles. Furthermore, all practically feasible care was observed by our researchers and animal care technicians to ensure that these mice received identical upbringing until our experiment. Hence, individual variation between animals with the same genotype is minimal. For statistical comparison of two genotypes, there is much less variability in the results and our sample sizes are sufficient.
Data exclusions	No animal or data point was excluded from analysis
Replication	All histology, calcium imaging, and electrophysiology experiments were repeated using tissues from at least 3 different mice. All attempts at replication were successful.
Randomization	Animals were placed into experimental groups based either on their genotype (no randomization) or through simple randomization.
Blinding	Behaviors were scored by researchers blinded to mouse genotypes or treatment condition.

Reporting for specific materials, systems and methods

We require information from authors about some types of materials, experimental systems and methods used in many studies. Here, indicate whether each material, system or method listed is relevant to your study. If you are not sure if a list item applies to your research, read the appropriate section before selecting a response.

Materials & experimental systems

n/a	Involved in the study	
☐	☒	Antibodies
☐	☒	Eukaryotic cell lines
☒	☐	Palaeontology
☐	☒	Animals and other organisms
☒	☐	Human research participants
☒	☐	Clinical data

Methods

n/a	Involved in the study	
☒	☐	ChIP-seq
☒	☐	Flow cytometry
☒	☐	MRI-based neuroimaging

Antibodies

Antibodies used	Primary antibodies used are: rabbit anti-TRPV1 (VR1-C; RA14113; Lot#401653, Neuromics, used at 1:1000 diluted), chicken anti-GFP (GFP-1020; Lot# GFP697986, Aves Lab; used at 1:1000 diluted). Secondary antibodies are: goat anti-rabbit IgG (Alexa Fluor-555; A21429, Lot# 1683674, Life technologies; used at 1:500 diluted), donkey anti-chicken IgG (FITC conjugated; 703-095-155, Lot#114050, Jackson ImmunoResearch; used at 1:500 diluted).
Validation	Validation data for antibodies are available from the commercial providers.

Eukaryotic cell lines

Policy information about cell lines

Cell line source(s)	KNRK cells obtained from ATCC
Authentication	The authentication data are available from ATCC

Mycoplasma contamination	The cell line was not tested for mycoplasma contamination in the authors' laboratory.
Commonly misidentified lines (See ICLAC register)	No commonly misidentified cell lines were used

Animals and other organisms

Policy information about studies involving animals; ARRIVE guidelines recommended for reporting animal research

Laboratory animals	C57BL/6J wild-type (Stock#: 000664), Trpm8-/- (Stock#: 008198), Trpv1-/- (Stock#: 003770), Rosa26-TrpV1 (Stock#: 008513), Tac1-/- (Stock#: 004103) mice were purchased from Jackson Laboratory. PirtGCaMP3/+ mice were gifted by Dr. Xinzhong Dong at Johns Hopkins University. Trpm8-eGFPf/+ mice were gifted by Dr. Gina Story when she worked at Washington University Pain Center. TrpM8-CreER/+ mice were from Dr. Hongzhen Hu at Washington University Center for the Study of Itch. All transgenic animals used for behavioral experiments were backcrossed to the C57BL/6 background, and all mice were between 2-3 months old at the time of experiment.
Wild animals	No wild animals were used in this study.
Field-collected samples	The study did not involve samples collected in the field.
Ethics oversight	All experimental procedures were approved by the Institutional Animal Care and Use Committee at Washington University in St. Louis, School of Medicine.

Note that full information on the approval of the study protocol must also be provided in the manuscript.
